# A Strategic Bargaining Game for a Spectrum Sharing Scheme in Cognitive Radio-Based Heterogeneous Wireless Sensor Networks

**DOI:** 10.3390/s17122737

**Published:** 2017-11-27

**Authors:** Yuxing Mao, Tao Cheng, Huiyuan Zhao, Na Shen

**Affiliations:** 1 School of Electrical Engineering, Chongqing University, Chongqing 400044, China; myx@cqu.edu.cn (Y.M.); zhaohy@cqu.edu.cn (H.Z.); 2 School of Nursing, Chengdu University of Traditional Chinese Medicine, Chengdu 611137, China; na_shen917@163.com

**Keywords:** wireless sensor networks, cognitive radio, strategic bargaining, spectrum sharing, Nash bargaining solution

## Abstract

In Wireless Sensor Networks (WSNs), unlicensed users, that is, sensor nodes, have excessively exploited the unlicensed radio spectrum. Through Cognitive Radio (CR), licensed radio spectra, which are owned by licensed users, can be partly or entirely shared with unlicensed users. This paper proposes a strategic bargaining spectrum-sharing scheme, considering a CR-based heterogeneous WSN (HWSN). The sensors of HWSNs are discrepant and exist in different wireless environments, which leads to various signal-to-noise ratios (SNRs) for the same or different licensed users. Unlicensed users bargain with licensed users regarding the spectrum price. In each round of bargaining, licensed users are allowed to adaptively adjust their spectrum price to the best for maximizing their profits. . Then, each unlicensed user makes their best response and informs licensed users of “bargaining” and “warning”. Through finite rounds of bargaining, this scheme can obtain a Nash bargaining solution (NBS), which makes all licensed and unlicensed users reach an agreement. The simulation results demonstrate that the proposed scheme can quickly find a NBS and all players in the game prefer to be honest. The proposed scheme outperforms existing schemes, within a certain range, in terms of fairness and trade success probability.

## 1. Introduction

With the development of Wireless Sensor Networks (WSNs), the radio spectrum resource, as one of the scarcest and expensive resources, gradually became a stumbling block. As wireless applications increase sharply, there are fewer and fewer unlicensed radio spectra, while licensed radio spectra are under-utilized. For unlicensed users lacking spectrum, solutions to the spectrum-sharing problem using dynamic spectrum borrowing and lending between unlicensed operators are inadequate [1]. Cognitive Radio (CR), which is considered to be a prospective approach to realize dynamic spectrum access (DSA) [2,3], is widely used to improve spectrum utilization. It allows licensed users’ resources to be efficiently shared with unlicensed users, in exchange for potential profits.

Usually, there are many alternative licensed users for a WSN. In a CR-based WSN [4,5,6], the spectrum demand for a licensed user directly depends on unit price and spectrum efficiency. To attract more buyers, licensed users need to compete with each other in the terms of spectrum price. In spectrum marketing, game theory provides a set of mathematical tools that can construct economic models to analyze the behaviors of self-interested users. Each type of game model, whether it is a cooperative or non-cooperative game, certainly has advantages and disadvantages [7,8,9,10]. Depending on different performances, their application environments are distinct.

On the basis of the Stackelberg game, the Distributed Optimization for Cognitive Radio (DOCR) scheme [11] was designed as a hierarchical framework to optimize CR network performance. In this scheme, the spectrum of a licensed user is divided into sub-bands, each of which can only be accessed by one unlicensed user. Hence, there is no difference in that all licensed users are regarded as one. In the work presented in this paper, it is unnecessary for users to communicate with each other. Using a simple pricing function for licensed users, a distributed algorithm is introduced in the DOCR scheme to converge on Stackelberg equilibrium. The multi-leader multi-follower Stackelberg (MMS) scheme [12] was proposed as an efficient spectrum sharing scheme. As leaders, multiple licensed users played a cooperative game and collaborated with each other to fairly distribute the outcome. As followers, multiple unlicensed users played a non-cooperative game using a self-interested strategy. During step-by-step iteration, a feedback learning process is repeated to find the best solution. Under widely-diverse network environments, the MMS scheme can find an effective solution to spectrum sharing, and offer an attractive performance balance. The two-stage resource allocation scheme, with combinatorial auction and Stackelberg game in spectrum sharing (TAGS) [13] provided a feasible solution to the spectrum sharing problem, and ensured all individuals’ economic properties. A geographically-restricted combinatorial auction, without consideration of spectrum recall, decided the spectrum allocations, while a Stackelberg game was designed to determine all users’ strategies with respect to the potential spectrum recall. However, the existence of the broker, which collected all sealed-bid information, determined the optimal allocation, and calculated the payments and payoffs, limited the framework of the model.

Recently, another game model, which is often used to solve spectrum sharing problems, is auction [14,15,16]. The Repeated Bayesian Auction (RBA) scheme [17] could achieve an effective solution of spectrum sharing under widely-diverse system environments. Licensed users adaptively decided their prices using a Bayesian game, and shared bandwidth based on the double auction protocol. In each auction round, the auctioneer collected all the information about offers and bids, and determined the trade price. The dynamic auction would be repeated sequentially, every time period, until an efficient auction consensus was reached. The Repeated Auctions with Bayesian Learning (RABL) scheme [18] investigated the problem of spectrum access in CR systems using monitoring and access costs. With incomplete information, a nonparametric belief update algorithm was proposed, based on the Dirichlet process. Although its convergence speed can be improved, it can achieve a good balance between efficiency and fairness. The Double Auction-based Spectrum Sharing (DASS) scheme [19] allows free spectrum band trading between operators to improve the efficiency of the spectrum utilization. The DASS scheme investigated the practical wireless communication framework using adaptive adjustable bidding/asking strategies.

Although an auction is an effective way to find a solution for multi-leader multi-follower spectrum sharing, the auctioneer is a limitation of practicability. Some other game models, for example, the Bertrand Game [20,21] and the evolutionary game [22], are presented to solve the multi-seller multi-buyer spectrum sharing problem. The Resource Pricing with Stackelberg Approach (RPSA) scheme [20] used the Bertrand Game to solve the resource allocation problem in CR networks. In addition, it introduced a control parameter to quantify the negative impacts. With the control parameter, the services of licensed users can be guaranteed. From the viewpoint of meeting the demand of network communication, the Bertrand Game based Spectrum Sharing (BGSS) scheme [21] was designed to solve the spectrum leasing and allocation problem. A flaw in the scheme is the principle that the spectrum requirements of unlicensed users must be satisfied. In the real world, an unlicensed user may choose not to trade if participating in the spectrum access game is deemed to be unfavorable.

To obtain the practicability and adaptability, the central controller (e.g., base station or auctioneer) cannot exist in the spectrum sharing model and there is no cooperation between licensed or unlicensed users. To deal with this matter, we designed a Strategic Bargaining based Spectrum Sharing (SBSS) scheme in heterogeneous WSNs (HWSNs). In HWSNs, unlicensed users have various signal-to-noise ratios (SNRs) for the same or different licensed users, which is more practical. Furthermore, to complete the spectrum trade between multiple licensed users and multiple unlicensed users, a non-cooperative strategic bargaining game was proposed to model the competition. It allows licensed and unlicensed users to adaptively decide their strategies. Without knowing other competitors’ information, each licensed and unlicensed user can carefully find the best strategy for themselves. Research regarding Nash bargaining [23,24] and HWSNs [8] is lacking; nonetheless, this paper proposes a scheme that outperforms existing schemes, as follows:

- In HWSN, not only are the differences (e.g., frequency band, bandwidth) between licensed users considered, but the discrepancies (e.g., hardware, space, and wireless environment) between unlicensed users are taken into account.
- Since there is no need to know competitors’ information, the non-cooperative game model no longer needs a central controller (base station or auctioneer). The advantages are that users in our model are adaptive and networks are distributed, which cannot be achieved by most existing schemes.
- The proposed scheme can be implemented in two scenarios according to the supply-and-demand relationship, i.e., less or more licensed radio spectrum supply than network demand.

In the rest of this paper, the system model, utility function, and strategic bargaining of the proposed scheme are described in Section 2. Section 3 presents a numerical performance analysis. Section 4 summarizes the work and provides a discussion.

## 2. Proposed Spectrum-Sharing Scheme

### 2.1. System Model

**Figure 1:**
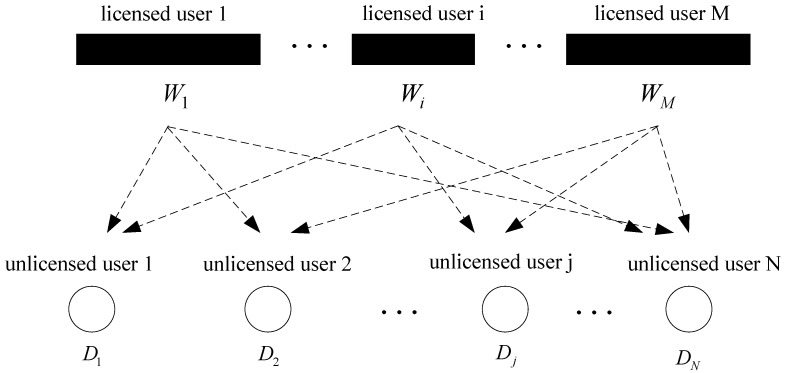
System model.

We consider a Cognitive Radio-based heterogeneous Wireless Sensor Network (CRHWSN) with N sensor nodes. The sensor nodes act as unlicensed users, purchasing idle spectra from M licensed users (Figure 1) without cooperation. Licensed user *i*, which has a certain amount of idle bandwidth, denoted by *W_i_*, can decide its spectrum price independently. Depending on the heterogeneous nature of the WSN, sensors are deposed under various environments and with discrepant hardware devices. Therefore, unlicensed users enjoy unequal SNR for one licensed user, and each unlicensed user has unequal SNR for different licensed users. With spectrum demand *D_j_*, unlicensed user *j* needs to decide which licensed user should be chosen, based on spectrum price and SNR. In this system model, all licensed users are non-cooperative and cannot achieve a coalition, which also applies to all unlicensed users.

### 2.2. Utility Function

To know how much profit users can obtain, we should find a way to quantify existing revenue and costs. For licensed user *i*, the profit function consists of three parts: Revenue from leasing spectrum to unlicensed users, revenue from providing service to ongoing licensed connections, and the cost induced by quality of service (Qos) degradation of the licensed user [25]. Therefore, the profit function of licensed user *i* can be formulated by:

(1) $$\begin{array}{c} \prod_{i}\left(P\right)=P_{i}\times \sum_{j=1}^{N}\left(D_{j}\times x_{ij}\right)+c_{1}\times M_{i}-c_{2}\times M_{i}\times {\left(B_{i}^{req}-k_{i}^{\left(l\right)}\frac{W_{i}-\sum_{j=1}^{N}\left(D_{j}\times x_{ij}\right)}{M_{i}}\right)}^{2}\\ st.\;\sum_{j=1}^{N}\left(D_{j}\times x_{ij}\right)\leq W_{i},\;i=1,\ldots ,M\\ \sum_{i=1}^{M}x_{ij}\leq 1,\;j=1,\ldots ,N\;\\ \;x_{ij}\in \left\{0,\;1\right\},\;i=1,\ldots ,M;\;j=1,\ldots ,N \end{array},$$

where *P* is the set containing all licensed users’ spectrum prices, that is, *P* = {*P_1_*, … *P_i_*, … *P_M_*}, *x_ij_* = 1 if unlicensed user *j* purchases spectrum from licensed user *i*; otherwise, *x_ij_* = 0, *M_i_* is the number of ongoing licensed connections, *c*_1_ and *c*_2_ denote the weights for the revenue and cost function, respectively; $B_{i}^{req}$ is the spectrum demand for an ongoing licensed connection; and $k_{i}^{\left(l\right)}$ is the spectral efficiency of wireless communication for licensed user i’s service provider, and can be obtained by:

(2) $$k_{i}^{\left(l\right)}=\frac{B_{i}^{req}}{W_{i}/M_{i}},$$

In this paper, we assume that once licensed user *i* decides to provide an unlicensed user with service, it will completely meet the spectrum demand of the unlicensed user. However, for an unprofitable market, an unlicensed user has the right to not choose any licensed user, namely, $\sum_{i=1}^{M}x_{ij}=0$. Related to the bandwidth, SNR, and spectrum price, the utility function of unlicensed user *j* is made up of the revenue gained from transmitting and the payment for radio resource usage. It can be calculated as follows:

(3) $$\begin{array}{ll} U_{j}(P)= & \omega_{j}\times D_{j}\times \sum_{i=1}^{M}\left(k_{ij}^{\left(u\right)}\times x_{ij}\right)-D_{j}\times \sum_{i=1}^{M}\left(x_{ij}\times P_{i}\right)\\ & st.\;\sum_{i=1}^{M}x_{ij}\leq 1,\;j=1,\ldots ,N\\ & x_{ij}\in \left\{0,\;1\right\},\;i=1,\ldots ,M;\;j=1,\ldots ,N \end{array},$$

where $\omega_{j}$ is the income from the per-transmission rate of unlicensed user *j*, and, in this paper, it also represents the importance of unlicensed user *j*’s data; $k_{ij}^{\left(u\right)}$ is the spectral efficiency of wireless communication by unlicensed user *j* using licensed user *i*’s spectrum, and can be obtained from Reference [26]:

(4) $$k_{ij}^{\left(u\right)}=\log_{2}\left(1+K\times \gamma_{ij}\right),\;\mathrm{where}\;K=\frac{1.5}{\ln\left(0.2/BER^{tar}\right)},$$

where $\gamma_{ij}$ is the SNR and is used to reflect the heterogeneous nature of WSN; *BER^tar^* is the target bit-error rate.

**Figure 2:**
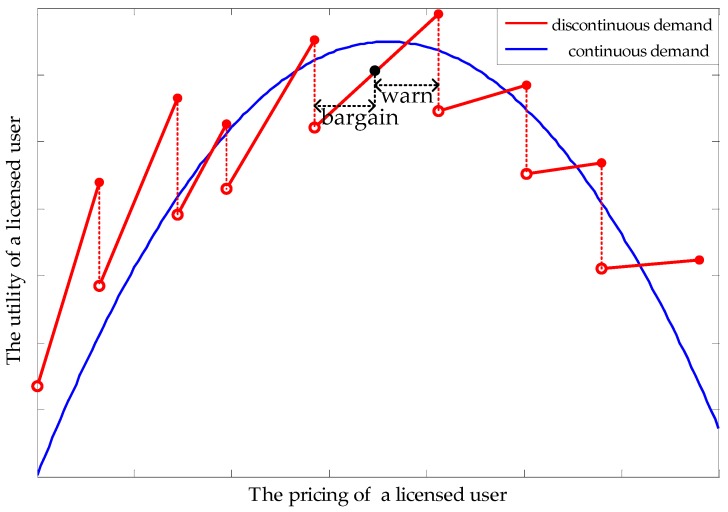
Utility function of a licensed user.

Some existing studies have proven that, when the spectrum demand from unlicensed users is continuous, the utility–price curve of a licensed user is continuous and strictly convex [20]. However, as shown in Figure 2, the discontinuous spectrum demands in this paper leads to discontinuous and straight utility-price lines. If the spectrum price varies within a limited range, the spectrum demand will not change, which is the reason why the utility of a licensed user is only proportional to its spectrum price. The spectrum price that reaches the peak point of a licensed user’s utility in a continuous situation no longer applies to a discontinuous situation. Note that, with an increase in spectrum price, each breakpoint means a reduction of buyers and the slope of each line decreases gradually.

### 2.3. Strategic Bargaining

Licensed users compete with each other, in terms of spectrum price, which will directly affect the strategies of unlicensed users. Once licensed users make their decisions, unlicensed users will buy spectra from whichever licensed user that will let them obtain maximum utility. Then, licensed users self-adaptively adjust their prices according to unlicensed users’ information, and unlicensed users again provide their best responses. These actions and reactions will either continue until some prescriptive deadline arrives or will end with an agreement. Of the many applications of strategic interactions, one ubiquitous situation naturally comes to mind: Strategic bargaining. We start the analyses with an easy case, in which only one round of bargaining is taken into consideration.

#### 2.3.1. One Round of Bargaining (the Ultimatum Game)

**Figure 3:**
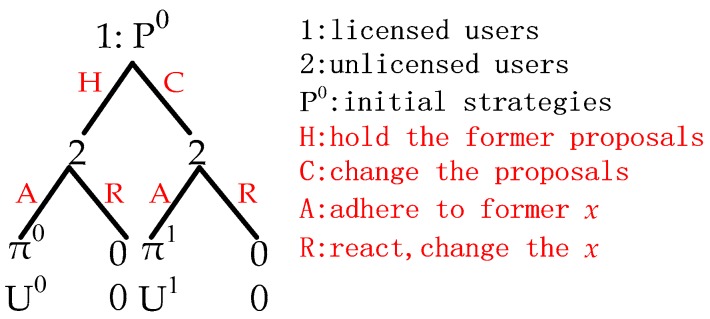
One round of bargaining (the ultimatum game).

Unlike classical one-round bargaining, in the ultimatum game [27], Player 1 or Player 2 (shown in Figure 3) represent two groups of users, rather than two players. In other words, each licensed user needs to complete the ultimatum game with all unlicensed users at the same time. If there are M licensed users, M ultimatum games will be carried out simultaneously. Formally, this paper uses one ultimatum game to represent this process for simplicity. Meanwhile, only if all users in player 1 choose to hold the former strategies will H (hold the former proposals) be obtained; otherwise, C (change the proposals) be the choice. Similarly, A (adhere to former *x*) will be achieved only if all unlicensed users adhere to their former choices (that is, *x* remains unchanged). Secondly, as a game of perfect information (unlicensed users know the proposals of licensed users before making a response), this paper not only let licensed users know unlicensed users’ choice (A or R (react, change the *x*)), but also know the “bargain” and “warn” from unlicensed users (Figure 2). For any *x_ij_* which equals 0, “bargain” means how much price licensed user *i* needs to reduce, where unlicensed user *j* will change to choose licensed user *i*. “Warn”, aiming at *x_ij_* = 1, tell the licensed user *i* the spectrum price, at which it will lose the buyer *j*. Knowing these bargains and warns, and having all the “bargaining power” (due to the first-mover advantage), each licensed user can find the optimal price.

Although the one round of bargaining in this paper is different from the classical one, all propositions in Chapter 11.1 of Reference [27] are also applicable. Therefore, it can also be divided into an ultimatum game. With respect to our game model, we performed some adaptations, proposed some new propositions, and make the corresponding proofs.

**Proposition** **1.** *In one round of the bargaining game, any proposal that makes the utility of Players 1 and 2 be “not less than zero”, can be supported as a Nash equilibrium*.

**Proof.**  *We suppose the utility of Players 1 and 2 to be π and U, respectively. No matter whether Player 1 chooses H or C, Player 2’s strategy will be “I accept any proposal making U ≥ 0 and reject any proposal making U < 0.” For Player 1, any strategy that makes π ≥ 0 will be adopted. Therefore, π ≥ 0 and U0 are the mutual best responses, and the proposition has been proven*. ☐

Although Player 1 will accept any strategy that makes π ≥ 0, whether there is a π that let Player 1 obtain the maximum utility under the premise of U ≥ 0, without cooperation, licensed users in Player 1 have no need, nor ability, to achieve a global optimum. On the contrary, the individual optimum of one licensed user is more meaningful. This expectation leads to the following proposition:

**Proposition** **2.** *One round of the bargaining game will have at least M Nash equilibria, if Player 1 has M licensed users*.

**Proof.**  *We assume that there is a situation where licensed user i obtains optimal utility under the premise of x remaining unchanged (it can be obtained if licensed user i adjusts the price according to “warn”, and other licensed users hold the former strategies). In this situation, if any other licensed user changes the strategy, the x will change and (C, R) will be obtained (as shown in Figure 3). If any unlicensed user changes the choice, x will also change and (H, R) or (C, R) will be obtained. Meanwhile, (H, R) or (C, R) will cause the utility of Players 1 and 2 to reduce to zero. In other words, other licensed and unlicensed users cannot obtain more benefits; the proposed situation is a Nash equilibrium. Because I ∈ {1, 2,…, M}, there are at least M Nash equilibria, which proves the proposition*. ☐

We have proved the existence of some Nash equilibria, but what we indeed need is at least one subgame-perfect equilibrium in strategic bargaining.

**Proposition** **3.** *Each M Nash equilibrium in Proposition 2 is neither a sequentially-rational Nash equilibrium, nor a subgame-perfect equilibrium. Furthermore, the bargaining game with a single round can own at least one subgame-perfect equilibrium, if each* $\gamma_{ij}$ *in* γ *has diversity.*

**Proof.**  *For the above M Nash equilibria, because all licensed users are sequentially rational, they all have the desire to obtain their own optimal utility instead of holding former strategies. Therefore, the M Nash equilibria are not sequentially optimal for all licensed users and are not subgame-perfect equilibria. The proof of the remaining part of Proposition 3 is explained in the following section*. ☐

#### 2.3.2. Finitely Many Rounds of Bargaining

Through one round of bargaining, the Nash equilibrium always can’t be found. Therefore, the bargaining will repeat once and once again until the Nash equilibrium is obtained. In practical applications, an infinite game cannot exist, depending on the exogenous deadline. With limited rounds of bargain, finitely many rounds of bargaining [27] can solve the spectrum sharing problem as follows:

(5) $$\begin{array}{c} \arg\underset{P_{i}}{\max}\;\Pi_{i}\left(P\right),\;for\;\forall i\in \left\{1,\;2,\;\ldots ,\;M\right\}\\ st.\;\sum_{j=1}^{N}\left(D_{j}\times x_{ij}\right)\leq W_{i},\;i=1,\ldots ,M\\ x_{ij}\in \left\{x_{ij}|\arg\underset{x_{ij}}{\max}\;U_{j}\left(P\right),\;for\;all\;i\in \left\{1,\;2,\;\ldots ,\;M\right\}\right\},\;j=1,\ldots ,N \end{array}.$$

**Figure 4:**
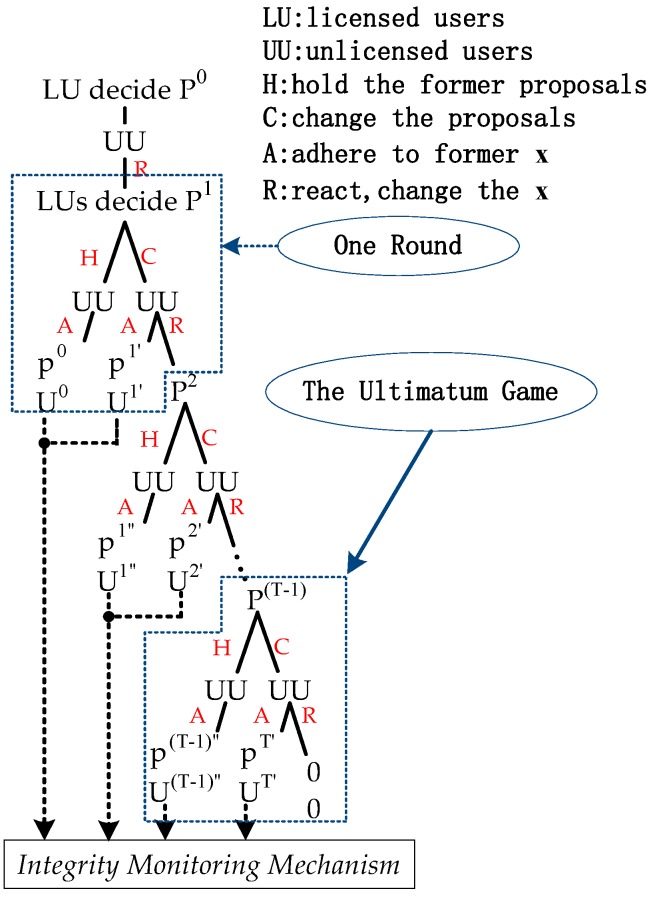
Finitely many rounds of bargaining.

In order to obtain its own optimal utility, each licensed user will dynamically adjust their spectrum price. Each time a user makes the strategic decision, it depends on the last action of the opponent. That is to say, each user in Player 1 updates their strategy, based on the previous spectrum demand from Player 2, while each user in Player 2 makes their choice according to Player 1’s price. A process, where all users can dynamically find the optimal strategies, will continue until a consensus is reached. This paper uses finitely many rounds of bargaining (Figure 4) for modeling. Because of the existence of a deadline, the ultimatum game is designed as the penultimate part of a strategic game. When licensed users hold the former proposals (namely, choose the H), the responses of unlicensed users are already optimal. Therefore, compared with the ultimatum game mentioned above, the ultimatum game in this part does not have (H, R). Regarding the existence of subgame-perfect equilibrium in the ultimatum game, as shown in Figure 4, we prove the Proposition 4.

**Proposition** **4.** *If the finitely many rounds of bargaining go to the ultimatum game, the (H, A) will be a subgame-perfect equilibrium*.

**Proof.**  *In the ultimatum game, if any licensed user selfishly changes their price in favor of more profit, without knowing other licensed users’ strategies in that round, there is a high probability that x will change. Obviously, those changes will lead to (C, R), which will cause all users to gain nothing. Therefore, for sequentially-rational users, (H, A) will be a subgame-perfect equilibrium. It is worth mentioning that one or more subgame-perfect equilibria will exist, which is corroborated using our many simulations*. ☐

Knowing the “bargain” or “warn” from all unlicensed users, each licensed user can draw a utility curve (as in Figure 2). From this curve, a licensed user can find an optimal price, which will allow unlicensed users to enter a new round of bargaining. This process will continue until strategies of licensed and unlicensed users no longer change, and a Nash bargaining solution is found. Because the subgame-perfect equilibrium in the ultimatum game is probably not optimal for licensed and unlicensed users, all users are willing to come to an agreement before the ultimatum game.

### 2.4. Integrity Monitoring Mechanism

As shown in Figure 4, once Players 1 and 2 come to an agreement, or the deadline is reached, Player 1 will add an additional round, which is an integrity monitoring mechanism. The integrity monitoring mechanism is prepared for dishonest unlicensed users, who will falsely report the “bargain” and “warn” as follows:

(6) $$\beta_{ij}=(1+\delta_{j})\times \beta_{ij},\;i=1,\ldots ,M;\;j=1,\ldots ,N,$$

(7) $$\varpi_{ij}=(1+\delta_{j})\times \varpi_{ij},\;i=1,\ldots ,M;\;j=1,\ldots ,N,$$

where $\beta_{ij}$ and $\varpi_{ij}$ are the “bargain” and “warn” from unlicensed user *j* to licensed user *i*, respectively, and *δ_j_* is the dishonesty degree of unlicensed user *j*, *δ_j_* ∈ [0, 1]. When *δ_j_* = 0, unlicensed user *j* is honest. The closer to 1 the value is, the more dishonest the user is. The reason why unlicensed users perform in this way is because all unlicensed users want licensed users to reduce their prices.

**Figure 5:**
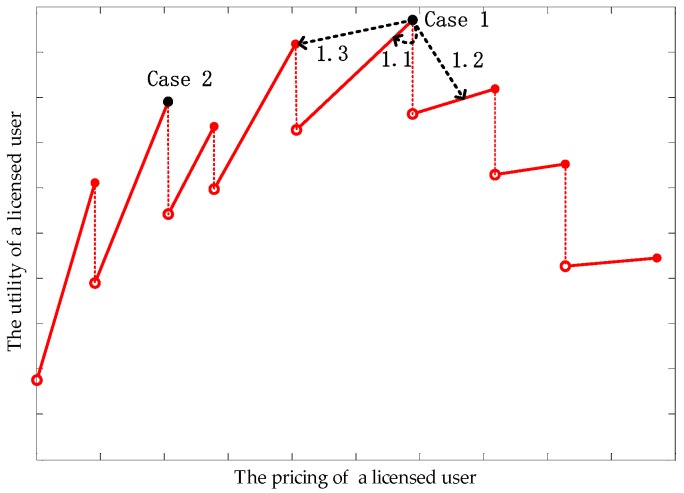
Cases of dishonest unlicensed user.

Before designing the integrity monitoring mechanism, we needed to analyze all the cases:

**Case** **1:** Single unlicensed user is dishonest: Located on the straight line with maximal utility; if they become dishonest, as shown in Figure 5, case 1.1, case 1.2, or case 1.3 will occur.
**Case** **2:** Single unlicensed user is dishonest: Not located on the straight line with maximal utility; therefore, their dishonesty cannot affect the licensed user’s strategy.
**Case** **3:** All unlicensed users are dishonest: The peak of each straight line will reduce, leading to a reduction in a licensed user’s utility.
**Case** **4:** Some unlicensed users are dishonest; this case can be analyzed using Cases 1, 2, and 3.

The probability of each case happening, and the utility of Players 1 and 2, are intuitively stated in Table 1. Case 1.1 causes a slight reduction in price, which leads to a slight reduction in π and an increase in U. Since the suboptimal price is usually lower, Case 1.2 will occur with a lower probability than Case 1.3. A dishonest unlicensed user choosing another licensed user will cause a new Nash bargaining solution (NBS). Normally, a new NBS will be a higher price, caused by the chain reaction of the corresponding licensed user increasing their price. The slight reduction in π in Case 1.3 is dependent on a suboptimal price, and a lower price results in an increase in U, while a higher price leads to a reduction. The reason why Case 2 happens with a high probability is that the majority of unlicensed users do not lie on the straight line of maximal utility. Non-cooperative unlicensed users cannot easily achieve Case 3 without collusion. Their different *δ* values make the U of each unlicensed user uncertain.

On the one hand, through analyzing these cases, we found that dishonest unlicensed users can make negligible profit with a very low probability. A rational unlicensed user has no desire to be dishonest. On the other hand, an integrity monitoring mechanism is also needed for irrational unlicensed users. Licensed users with the first-mover advantage can add an additional integrity monitoring round, whenever an optimal proposal is found. It is worth noting that the mechanism is aimed at unlicensed users in Case 1. In the integrity-monitoring round, each licensed user slightly increases their price. If the choice of the unlicensed user changes, the conclusion that this unlicensed user is honest will be drawn; otherwise, the unlicensed user is dishonest. After the monitoring round, the price is restored to its original value and the dishonest, unlicensed user cannot buy any spectra from the licensed user. Without knowing of the approach of the monitoring round, dishonest unlicensed users cannot pretend to be honest.

## 3. Results

We consider a network model with four licensed users, providing service for a HWSN, which has fifteen sensors as unlicensed users. All simulations are done in MATLAB 2014a. Other parameter settings can be seen in Table 2.

### 3.1. Nash Bargaining Solution

**Figure 6:**
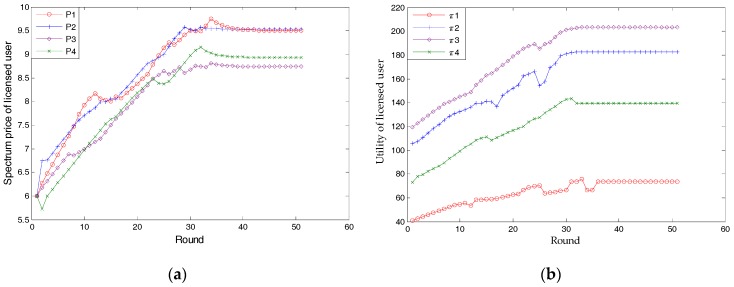
The process of finding Nash bargaining solution (NBS): (**a**) spectrum price; (**b**) utility of licensed user.

Figure 6 simulates the finitely many rounds of bargaining in Figure 4. In the 51st round, the proposed strategic game finds a Nash bargaining solution, namely, a subgame-perfect equilibrium, where the spectrum price is [9.50, 9.53, 8.74, 8.93], the utility of a licensed user is [73.67, 182.84, 203.26, 139.79], and the sold bandwidth of a licensed user is [5, 23, 28, 17]. Licensed user 1 sells spectra to unlicensed user 3; licensed user 2 supplies service to unlicensed users 2, 4, 8, 11, and 14; licensed user 3 sells spectra to unlicensed users 5, 10, 12, 13 and 15; and licensed user 4 satisfies the spectra of unlicensed users 1, 6, and 9. Noted that, unlicensed user 7’s demand has not been met. This is because its *k*^(*u*)^ is so small that its utility is negative. The principle of the proposed scheme is that unlicensed users will not buy spectra when the utility is negative. On the other hand, this result also shows that the proposed scheme will not always meet the spectrum demands of unlicensed users.

**Figure 7:**
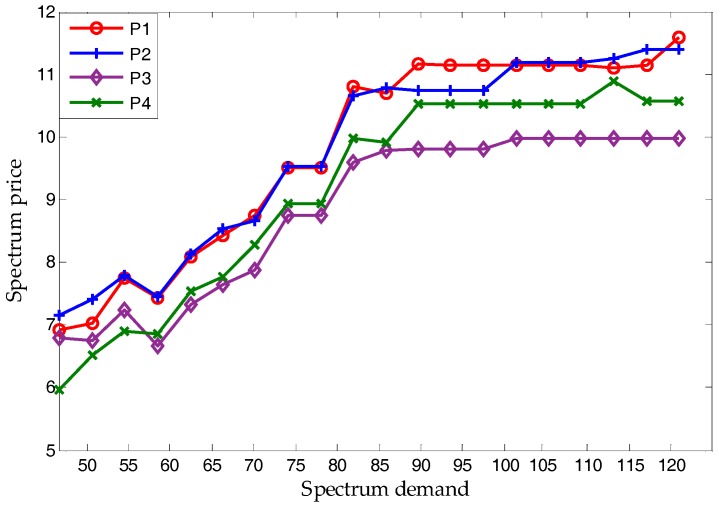
The relationship between NBS (spectrum price) and spectrum demand.

By analyzing *k*^(*u*)^, we find that if we only consider the *k*^(*u*)^ of each unlicensed user, the spectrum demands of licensed users 1 and 2 will appear to be greater than they are. Therefore, to gain maximal utility, licensed users 1 and 2 need to reduce buyers by using a higher price, while licensed users 3 and 4 need to use a relatively lower price to attract customers. The influence of the bandwidth of the licensed user can also not be ignored. Owing to relatively lower bandwidth availability, licensed user 1 has the highest price; on the other hand, licensed user 3 has the lowest price. Figure 7 proves licensed user 3’s principle of small profits, yet quick turnovers. With a higher price than licensed user 1, licensed user 2 sells less bandwidth but gains almost the same profits.

As mentioned before, spectrum price will converge on the NBS. Figure 7 shows that, except for *k*^(*u*)^ and licensed users’ bandwidth, the supply-and-demand relationship is also a vital factor. When the spectrum demand of HWSNs range from 50 to 120 (the maximal supply of licensed users is 100), the spectrum price of all licensed users will increase along with it. Due to the character of each licensed user and unlicensed users’ SNR, the relative relationship of licensed users’ prices remain almost the same. Note that, no matter what the relationship between supply and demand may be, Figure 7 proves the proposed scheme to be applicable.

### 3.2. The Influence of Dishonest Unlicensed Users

**Figure 8:**
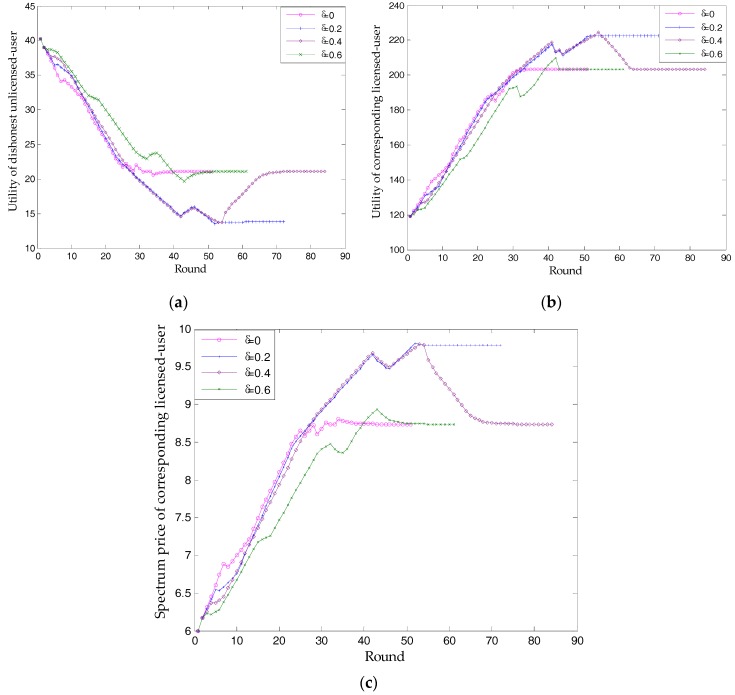
The influence of single unlicensed-user being dishonest: (**a**) utility of itself; (**b**) utility of corresponding licensed user; (**c**) spectrum price of corresponding licensed user.

In this paper, we assume that unlicensed user 15 is dishonest. When it acts as a dishonest user, Figure 8 shows that it can make extra profits in the process of bargaining. The more dishonest it is, the greater its profits are. However, in most cases (dishonesty degree is 0, 0.4, and 0.6), the utility of the unlicensed user and the corresponding licensed-user will, respectively, converge on the same value (U = 21.1, π = 203.3), and that the spectrum price and choices of all unlicensed users also remain the same. This is proof of the high probability of Case 2. As can be seen in Figure 8, dishonesty causes longer bargaining times (shown by rounds). Therefore, a dishonest unlicensed user selects a high risk of making no profit and meeting the deadline. Each rational unlicensed user will have no desire to cheat.

The phenomenon, in which the curve in Figure 8a and corresponding curve in Figure 8b form a trend of complementing each other, is interesting. This is because, for one side of a trade, generating profits is always at the expense of the other’s revenue. Another phenomenon, which is unusual, is seen when the dishonesty degree is 0.2. In order to determine why U decreases to 13.8, while π increases to 222.7, we performed further simulations. Regarding spectrum price, Figure 8c indicates that, in the process of bargaining, price converges on a higher value. In fact, the spectrum prices of all licensed users increase from [9.5, 9.53, 8.74, 8.93] to [10.3, 10.33, 9.78, 9.49], which directly lead to a higher π and a lower U. Although this process is difficult, it can also be explained by Case 1.2. In one round of bargaining, dishonesty causes a higher price for licensed user 3, which causes an increase in other licensed-users’ prices. Owing to this general increase in price, unlicensed user 13 no longer chooses any licensed user. The proposed scheme finds another NBS of this market.

**Figure 9:**
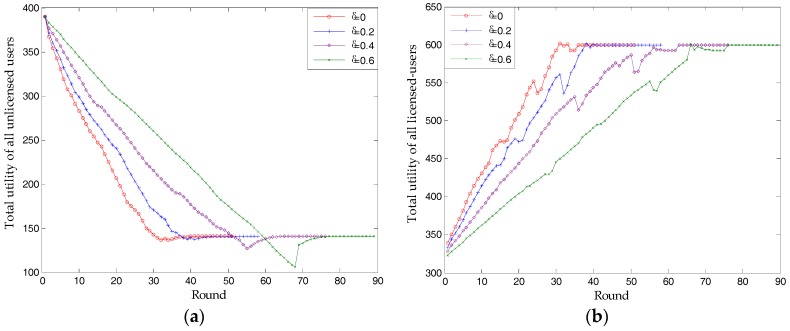
The influence of all unlicensed users being dishonest: (**a**) utility of all unlicensed users; (**b**) utility of all licensed users.

Having analyzed the influence of a single unlicensed user being dishonest, Figure 9 simulates Case 3. Unexpectedly, although the dishonesty degree of all unlicensed-users increases, the total utility of licensed and unlicensed users both remains the same (π = 599.6, U = 141.0). Further, we find that the proposed scheme can always find the same optimal price ([9.5, 9.53, 8.74, 8.93]), no matter how dishonest all unlicensed users are. However, the analysis in Table 1 shows that the total utility of licensed users will reduce. As mentioned in Figure 7, the supply-and-demand relationship is essential in deciding NBS. Although all unlicensed users are cheating, their spectrum demands have no differences. Since the analyses in Table 1 are based on a situation where dishonest unlicensed users occur after a NBS is found, the analyses in Table 1 are also correct. In other words, the supply-and-demand relationship deciding NBS, can be achieved in the process of many rounds of bargaining, before, rather than after, a NBS is found.

Figure 9 also shows that a greater dishonesty degree can only let unlicensed users make more profits in the process of finding NBS.

### 3.3. Other Simulations

**Figure 10:**
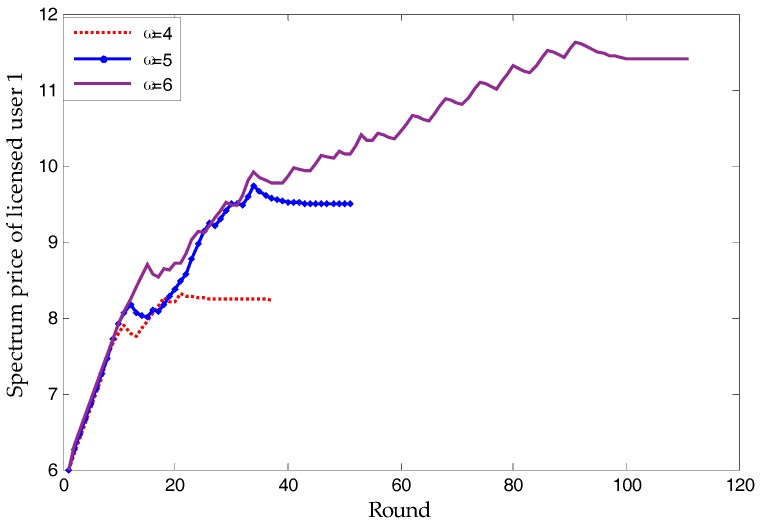
The relationship between spectrum price and ω.

The conclusion that a greater *ω* brings about a higher price can be drawn from Figure 10. From Equation (3), a greater *ω* means that, to achieve an equal U, a higher price is needed. To make the curve more clear, other licensed users’ curves are not shown in Figure 10. As a matter of fact, other licensed users’ curves follow the same rules as licensed user 1, when *ω*_1_ changes. Generally speaking, unlicensed users with important data can bear a higher spectrum price.

**Figure 11:**
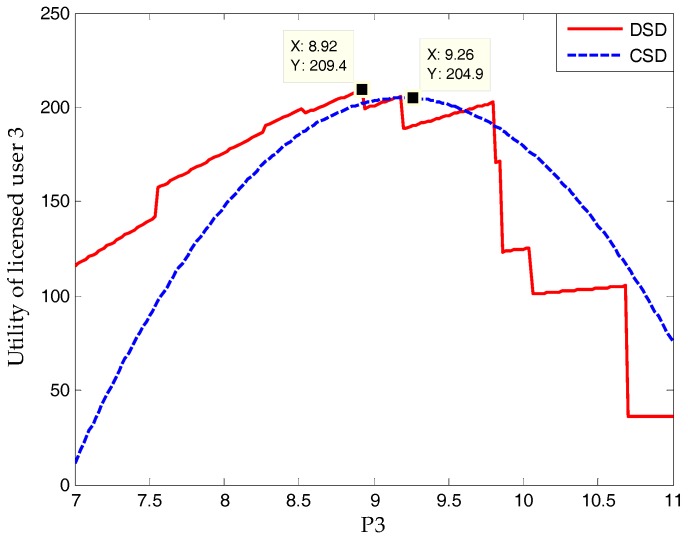
The discontinuous spectrum demand’s influence on licensed user’s utility.

The simulation in Figure 11 is to verify the hypothesis in Figure 2. As Figure 2 shows, a discontinuous spectrum demand (DSD) will lead to discontinuous straight lines of a licensed user’s utility. When comparing the utility curve with a continuous spectrum demand (CSD), the utility curve with a DSD obtains a different peak at a different price. In contrast to Figure 2, Figure 11 shows that, at the beginning of prices increasing, a reduction in spectrum demand does not cause a lower utility in the licensed user. This is because the cost of Qos degradation greatly decreases, which leads to a higher utility.

**Figure 12:**
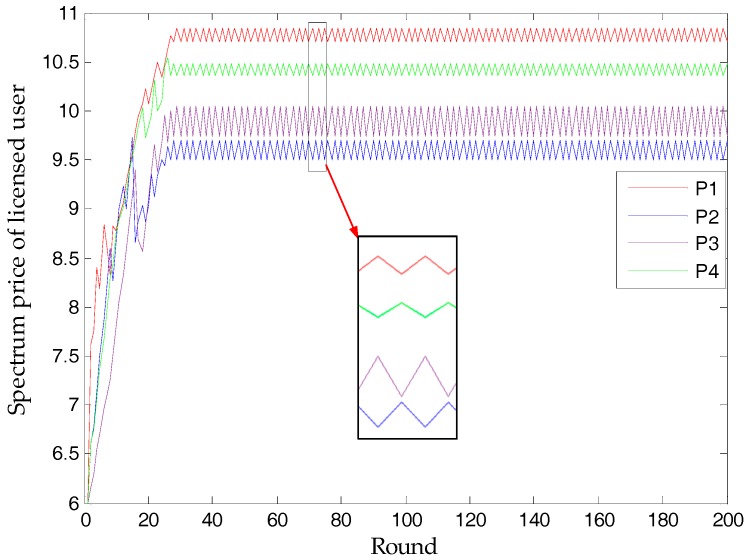
The existence of several subgame-perfect equilibria.

By randomly generating *k*^(*u*)^, we get a simulation result, as in Figure 12. In fact, this proves that a bargaining game can own at least one subgame-perfect equilibrium, as proposed in Proposition 3. In this simulation, there are two subgame-perfect equilibria. Although several subgame-perfect equilibria cannot allow both sides to come to an agreement, our algorithm adopts the last one proposed in the ultimatum game. Similarly, no matter how many subgame-perfect equilibria there are, the proposed scheme can determine one to be the final NBS.

### 3.4. Comparison with Existing Schemes

As the existing pricing-based spectrum-sharing schemes have differences in the network model and in utility function, it is difficult to compare them with our scheme. Nonetheless, we compare the performance in terms of trade success probability and fairness. All measures are based on 50 simulation runs. The trade-success probability of the proposed scheme is the probability of finding a unique NBS before the ultimatum game. Network fairness (*F*) is calculated based on the Jain’s fairness index [28], as follows:

(8) $$F={\left(\sum_{i=1}^{M}\sum_{j=1}^{N}\left(D_{j}\times x_{ij}\right)\right)}^{2}/\left(M\times {\sum_{i=1}^{M}\left(\sum_{j=1}^{N}\left(D_{j}\times x_{ij}\right)\right)}^{2}\right),$$

where *F* ranges from 0 to 1. To obtain the network fairness, this paper reset the bandwidth to an equal value.

**Figure 13:**
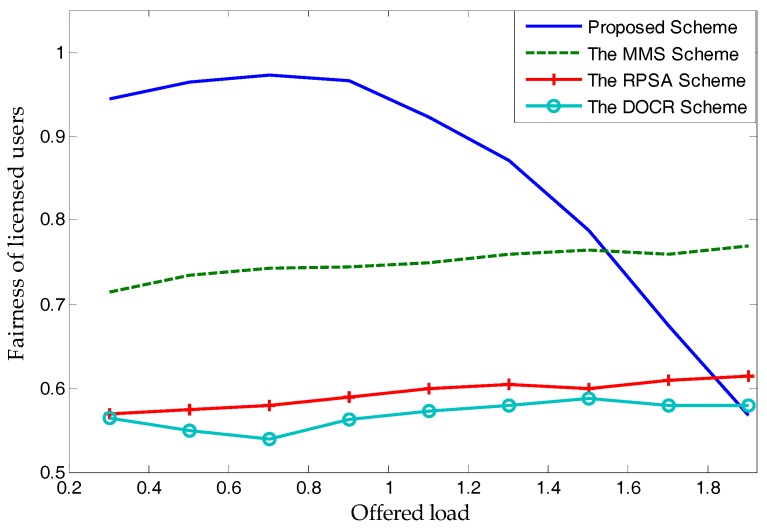
Network fairness.

In Figure 13, we compare the performance of the proposed scheme with three published schemes: The MMS scheme [12], RPSA scheme [20], and DOCR scheme [11], in terms of network fairness. As various SNRs decide different spectrum demands for a licensed user (different demands lead to the differences for each licensed user), an obvious reduction in fairness occurs when the offered load (service request rate, i.e., spectrum demand divided by spectrum supply) is more than 1. Nonetheless, when the offered load is less than 1.5, the proposed scheme has an absolute advantage.

**Figure 14:**
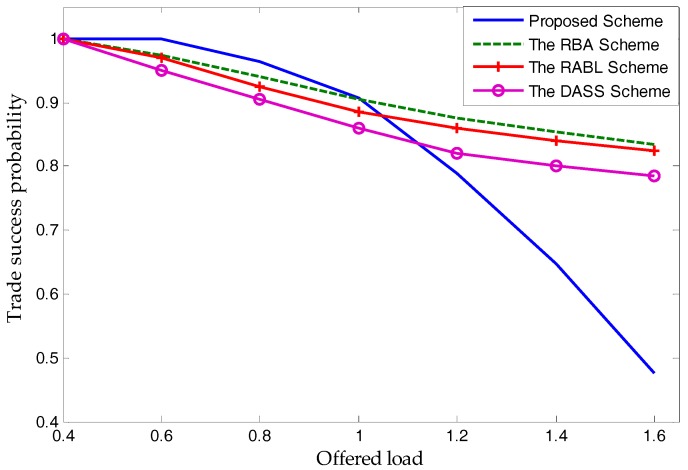
Trade success probability.

When compared with the proposed scheme with three schemes: the RBA scheme [17], the RABL scheme [18], and the DASS scheme [19], in terms of the trade success probability, Figure 14 shows that the proposed scheme has the highest probability when the offered load is less than 1. The existence of the ultimatum game, does not mean that the proposed scheme cannot apply to a case where the offered load is greater than 1.

Note that the results shown in Figure 13 and Figure 14 indicate that only the proposed scheme has a non-linear performance. This depends on the rules that spectrum price be directly related to the supply-and-demand relationship, and that unlicensed users buy spectra only when spectrum price leads to a non-negative utility. Different from existing schemes, the proposed scheme allows an unlicensed user to not buy spectra in this period for a positive utility. Therefore, when the offered load is greater than a certain value (almost 1), the rate of unlicensed users choosing to buy spectra will reduce significantly. Of course, it will reduce the trade success probability and fairness more substantially than other schemes. Therefore, as shown in Figure 13 and Figure 14, when other schemes have an approximately linear performance, the proposed scheme cannot be obtained.

## 4. Discussion

In terms of simulation, Figure 6 and Figure 12 verify Proposition 3, that the proposed scheme can own at least one subgame-perfect equilibrium for SNRs with diversity. Except for the dishonesty degree (in Figure 8) and the importance coefficient of unlicensed users’ data (in Figure 10), Figure 7 and Figure 9 directly or indirectly prove that the supply-and-demand relationship is the determining factor for a subgame-perfect equilibrium (namely, NBS). However, there are two NBSs without any changing parameters. In fact, the different NBSs in Figure 12 have different choices of unlicensed users, which have indirectly changed spectrum demand. In contrast, Figure 8 and Figure 9, not only prove the analyses in Table 1, but also provide evidence that both sides of the game have no desire to be dishonest. In daily life, the “bargain” is a common phenomenon, while the “warn” is an extra requirement in this paper. For our bargaining model, the “warn” seems to be unreasonable; however, it is necessary in order to know all information at the discontinuous point in Figure 11. With a discontinuous spectrum demand, a licensed user can no longer speculate the optimal strategy (not having the “bargain” and “warn”), which can be achieved by previous strategies if the spectrum demand is continuous.

All simulations prove that the proposed bargaining game can find a spectrum-sharing scheme, regardless of the HWSN. It can also be applied to various HWSNs with diverse spectra demands. If we take the fairness and trade success probabilities into consideration, a HWSN in which the offered load is less than 1.5 will be a good choice.

## Figures and Tables

**Table 1:** Analyses of all cases.

	Features	Probability	(π, U)
Cases			
Case 1	1.1	Low	(↓, $\overset{—}{↑}$)
	1.2	Lower	(?, ↓)
	1.3	Low	(↓, ↑)
Case 2		High	(—, —)
Case 3		Lowest	(↓, ？)

↓/↑: reduce/increase; ↓/$\overset{—}{↑}$: reduce/increase slightly; —: keep invariant; ?: be uncertain.

**Table 2:** Parameter settings.

M	the number of licensed users	$k^{\left(u\right)}$	the spectral efficiency of wireless communication by unlicensed user [2.063, 1.833, 1.726, 2.403 1.822, 2.129, 1.827, 1.964 2.229, 2.031, 1.994, 1.614 1.705, 2.285, 1.752, 1.782 2.182, 2.268, 2.305, 1.935 1.700, 2.147, 1.712, 2.105 1.900, 1.906, 1.747, 1.786 2.131, 2.280, 1.684, 2.112 2.256, 2.051, 1.749, 2.202 1.577, 1.878, 1.961, 1.742 2.367, 2.374, 1.838, 1.622 2.253, 2.329, 2.363, 1.823 2.009, 2.066, 1.956, 1.846 1.961, 2.129, 1.701, 1.950 1.972, 2.098, 2.350, 2.028]
	4		
N	the number of unlicensed users		
	15		
W/MHz	the bandwidth of each licensed users		
	[12, 28, 36, 24] (total: 100)		
*M’*	the number of ongoing licensed connections		
	[6, 14, 18, 12]		
$B^{req}$/MHz	the spectrum demand for an ongoing licensed connection		
	[2, 2, 2, 2]		
P^0^	the initial spectrum price		
	[6, 6, 6, 6]		
P^1^/P^2^/P^3^/P^4^	the spectrum price of licensed user 1/2/3/4		
*δ*	the dishonesty degree of unlicensed user		
$BER^{tar}$	the target bit-error rate		
	0.0001		
$c_{1}/c_{2}$	the weights for revenue/cost function		
	2/2		
ω	the income from the per-transmission rate of the unlicensed user		
	[5, 5, 5, 5, 5, 5, 5, 5, 5, 5, 5, 5, 5, 5, 5]		
D/MHz	the spectrum demand of unlicensed user		
	[2, 4, 5, 8, 3, 9, 5, 2, 6, 6, 3, 7, 5, 6, 7]		

Because $\gamma_{ij}\in \left[9,\;22\right]$, $k_{ij}^{\left(u\right)}\in [1.4731,\;2.4173]$ and is randomly generated.
